# Supplementary material for: All-small-molecule organic solar cells with over 14% efficiency by optimizing hierarchical morphologies
Source: Nat Commun. 2019 Nov 26;10:5393. doi: 10.1038/s41467-019-13292-1 (PMC6879588; doi:10.1038/s41467-019-13292-1)
Supplement: Supplementary file 1 — Supplementary Information [file 41467_2019_13292_MOESM1_ESM.pdf]

Supplementary Information

for

All Small Molecule Organic Solar Cells with Over 14% Efficiency by Optimizing Hierarchical Morphologies

Ruimin Zhou[1,2,3,4], Zhaoyan Jiang[1,2], Chen Yang[1,2], Jianwei Yu[5], Jirui Feng[6], Muhammad Abdullah Adil[1,2], Dan Deng[1], Wenjun Zou[1], Jianqi Zhang[1], Kun Lu[1], Wei Ma[6],* Feng Gao[5],* and Zhixiang Wei[1,2]**

[1]CAS key laboratory of nanosystem and hierarchical fabrication, CAS Center for Excellence in Nanoscience, National Center for Nanoscience and Technology, Beijing 100190, China.

[2]University of Chinese Academy of Sciences, Beijing 100049, China.

[3]Sino-Danish Center for Education and Research, Sino-Danish College, University of Chinese Academy of Sciences, Beijing 100190, China.

[4]Nano-Science Center & Department of Chemistry, University of Copenhagen, Copenhagen DK-2100, Denmark

[5]Department of Physics Chemistry and Biology (IFM), Linköping University, Linköping SE-58183, Sweden.

[6]State Key Laboratory for Mechanical Behavior of Materials, Xi'an Jiaotong University, Xi'an 710049, China.

These authors contributed equally: Ruimin Zhou, Zhaoyan Jiang

Correspondence and requests for materials should be addressed to K.L. (email: lvk@nanoctr.cn) or to W.M. (email: msewma@xjtu.edu.cn) or to F.G. (email: feng.gao@liu.se) or to Z.W. (email: weizx@nanoctr.cn)

Supplementary Figures

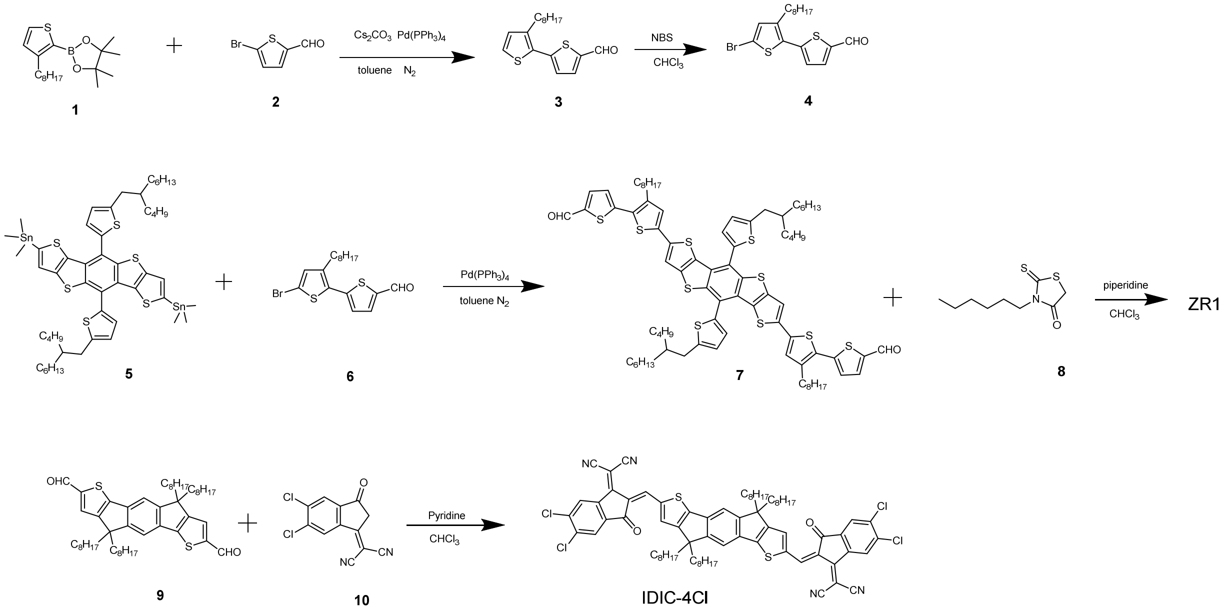

Supplementary Figure 1. Synthetic routes of ZR1 and IDIC-4Cl (The details of above molecules are given in the Supplementary Methods).

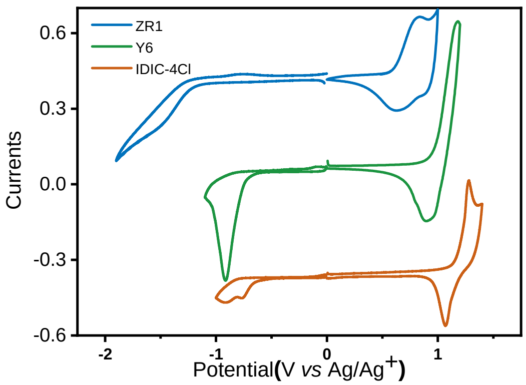

Supplementary Figure 2. Cyclic voltammogram (CV) plots of ZR1 (blue), Y6 (green) and IDIC-4Cl (red) in films.

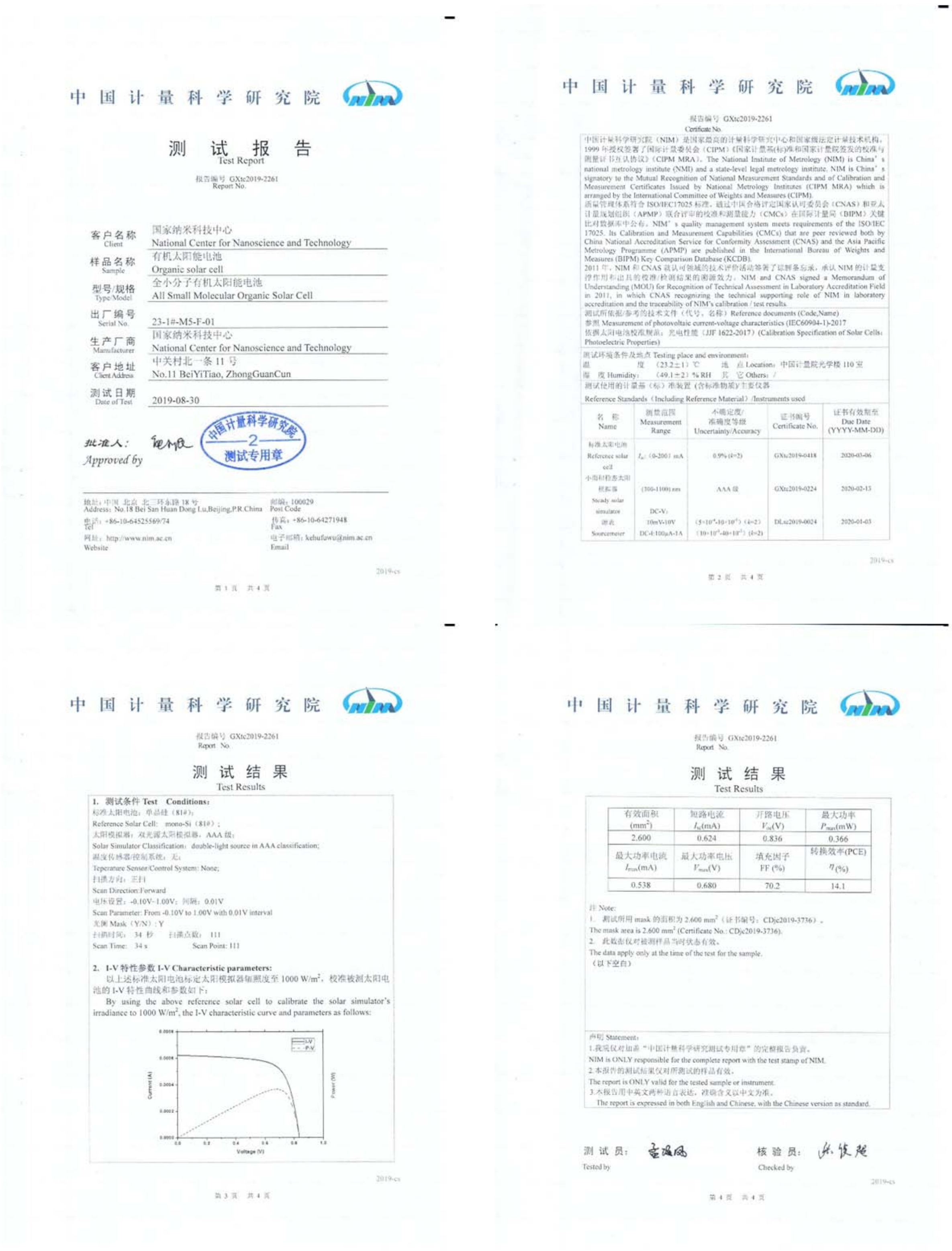

中国计量科学研究院

测 试 报 告
Test Report

报告编号 GXtc2019-2261
Report No.

客户名称 Client	国家纳米科技中心<br>National Center for Nanoscience and Technology
样品名称 Sample	有机太阳能电池<br>Organic solar cell
型号/规格 Type/Model	全小分子有机太阳能电池<br>All Small Molecular Organic Solar Cell
出厂编号 Serial No.	23-1#-M5-F-01
生产厂商 Manufacturer	国家纳米科技中心<br>National Center for Nanoscience and Technology
客户地址 Client Address	中关村北一条 11 号<br>No.11 BeiYiTiao, ZhongGuanCun
测试日期 Date of Test	2019-08-30

批准人: Approved by

中国计量科学研究院 2 测试专用章

地址：中国 北京 北三环东路 18 号
Address: No.18 Bei San Huan Dong Lu,Beijing,P.R.China
邮编：100029 Post Code
电话：+86-10-64525569/74 Tel
传真：+86-10-64271948 Fax
网址：http://www.nim.ac.cn Website
电子邮箱：kehufuwu@nim.ac.cn Email

2019-cs

第 1 页 共 4 页

中国计量科学研究院

报告编号 GXtc2019-2261
Certificate No.

中国计量科学研究院（NIM）是国家最高的计量科学研究中心和国家级法定计量技术机构，1999 年授权签署了国际计量委员会（CIPM）《国家计量基(标)准和国家计量院签发的校准与测量证书互认协议》（CIPM MRA）。The National Institute of Metrology (NIM) is China' s national metrology institute (NMI) and a state-level legal metrology institute. NIM is China' s signatory to the Mutual Recognition of National Measurement Standards and of Calibration and Measurement Certificates Issued by National Metrology Institutes (CIPM MRA) which is arranged by the International Committee of Weights and Measures (CIPM).
质量管理体系符合 ISO/IEC17025 标准，通过中国合格评定国家认可委员会（CNAS）和亚太计量规划组织（APMP）联合评审的校准和测量能力（CMCs）在国际计量局（BIPM）关键比对数据库中公布。NIM' s quality management system meets requirements of the ISO/IEC 17025. Its Calibration and Measurement Capabilities (CMCs) that are peer reviewed both by China National Accreditation Service for Conformity Assessment (CNAS) and the Asia Pacific Metrology Programme (APMP) are published in the International Bureau of Weights and Measures (BIPM) Key Comparison Database (KCDB).
2011 年，NIM 和 CNAS 就认可领域的技术评价活动签署了谅解备忘录，承认 NIM 的计量支撑作用和出具的校准/检测结果的溯源效力。NIM and CNAS signed a Memorandum of Understanding (MOU) for Recognition of Technical Assessment in Laboratory Accreditation Field in 2011, in which CNAS recognizing the technical supporting role of NIM in laboratory accreditation and the traceability of NIM's calibration / test results.

测试所依据/参考的技术文件（代号、名称）Reference documents (Code,Name)
参照 Measurement of photovoltaic current-voltage characteristics (IEC60904-1)-2017
依据太阳电池校准规范：光电性能（JJF 1622-2017）(Calibration Specification of Solar Cells: Photoelectric Properties)

测试环境条件及地点 Testing place and environment:
温 度 (23.2±1) ℃ 地 点 Location: 中国计量院光学楼 110 室
湿 度 Humidity: (49.1±2) %RH 其 它 Others: /

测试使用的计量器（标）准装置（含标准物质）/主要仪器
Reference Standards (Including Reference Material) /Instruments used

名 称 Name	测量范围 Measurement Range	不确定度/准确度等级 Uncertainty/Accuracy	证书编号 Certificate No.	证书有效期至 Due Date (YYYY-MM-DD)
标准太阳电池 Reference solar cell	I_{sc}: (0-200) mA	0.9% (k=2)	GXtc2019-0418	2020-03-06
小面积稳态太阳模拟器 Steady solar simulator	(300-1100) nm	AAA 级	GXtc2019-0224	2020-02-13
源表 Sourcemeter	DC-V: 10mV-10V<br>DC-I: 100μA-1A	$(5\times10^{-4}\cdot10\times10^{-4})$ (k=2)<br>$(10\times10^{-4}\cdot40\times10^{-3})$ (k=2)	DLxz2019-0024	2020-01-03

2019-cs

第 2 页 共 4 页

中国计量科学研究院

报告编号 GXtc2019-2261
Report No.

测 试 结 果
Test Results

1. 测试条件 Test Conditions:
标准太阳电池：单晶硅（81#）；
Reference Solar Cell: mono-Si（81#）;
太阳模拟器：双光源太阳模拟器，AAA 级；
Solar Simulator Classification: double-light source in AAA classification;
温度传感器/控制系统：无；
Teperature Sensor/Control System: None;
扫描方向：正扫
Scan Direction:Forward
电压设置：-0.10V-1.00V；间隔：0.01V
Scan Parameter: From -0.10V to 1.00V with 0.01V interval
光阑 Mask（Y/N）: Y
扫描时间：34 秒 扫描点数：111
Scan Time: 34 s Scan Point: 111

2. I-V 特性参数 I-V Characteristic parameters:
以上述标准太阳电池标定太阳模拟器辐照度至 1000 W/m²，校准被测太阳电池的 I-V 特性曲线和参数如下：
By using the above reference solar cell to calibrate the solar simulator's irradiance to 1000 W/m², the I-V characteristic curve and parameters as follows:

2019-cs

第 3 页 共 4 页

中国计量科学研究院

报告编号 GXtc2019-2261
Report No.

测 试 结 果
Test Results

有效面积 (mm²)	短路电流 I_{sc}(mA)	开路电压 V_{oc}(V)	最大功率 P_{max}(mW)
2.600	0.624	0.836	0.366
最大功率电流 I_{max}(mA)	最大功率电压 V_{max}(V)	填充因子 FF (%)	转换效率(PCE) η(%)
0.538	0.680	70.2	14.1

注 Note:
1. 测试所用 mask 的面积为 2.600 mm²（证书编号：CDjc2019-3736）。
The mask area is 2.600 mm² (Certificate No.: CDjc2019-3736).
2. 此数据仅对被测样品当时状态有效。
The data apply only at the time of the test for the sample.
（以下空白）

声明 Statement:
1.我院仅对加盖"中国计量科学研究院测试专用章"的完整报告负责。
NIM is ONLY responsible for the complete report with the test stamp of NIM.
2.本报告的测试结果仅对所测试的样品有效。
The report is ONLY valid for the tested sample or instrument.
3.本报告用中英文两种语言表达，释疑含义以中文为准。
The report is expressed in both English and Chinese, with the Chinese version as standard.

测试员: Tested by 核验员: Checked by

2019-cs

第 4 页 共 4 页

Supplementary Figure 3. Certification of the photovoltaic efficiency of the ZR1: Y6 devices by National Institute of Metrology, China (NIM).

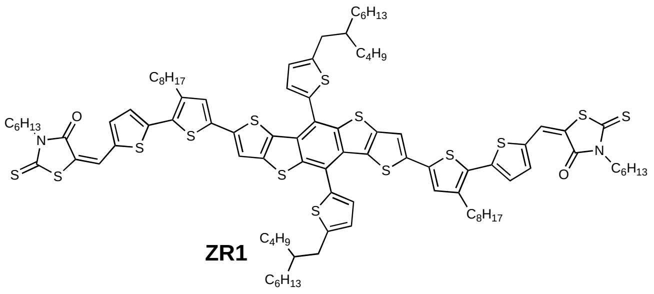

ZR1

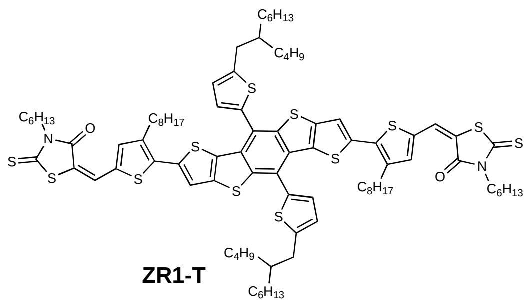

ZR1-T

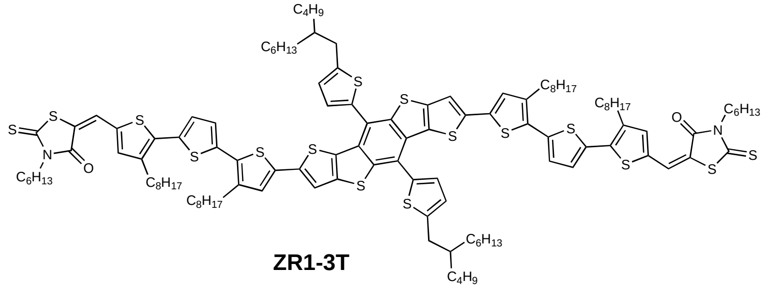

ZR1-3T

Supplementary Figure 4. ZR1, ZR1-T, ZR1-3T molecular structures.

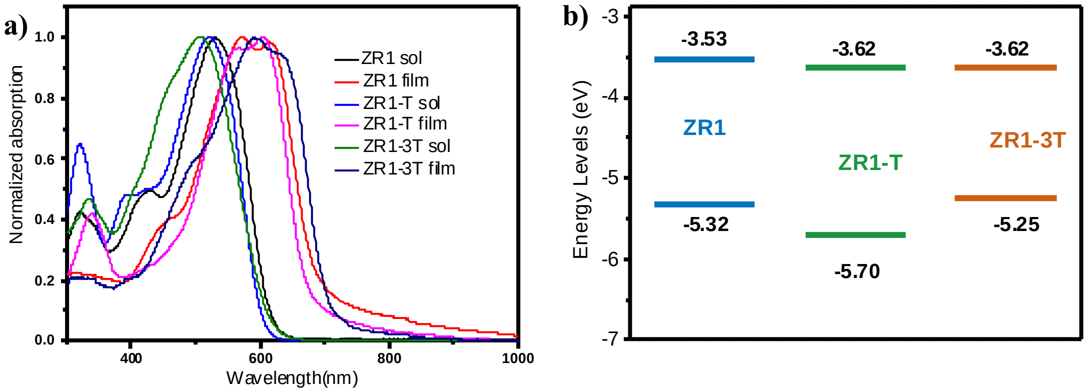

Supplementary Figure 5. a) Normalized UV–vis absorption spectra of ZR1, ZR1-T, ZR1-3T in solution and thin films. **b)** Energy diagrams of ZR1, ZR1-T, ZR1-3T

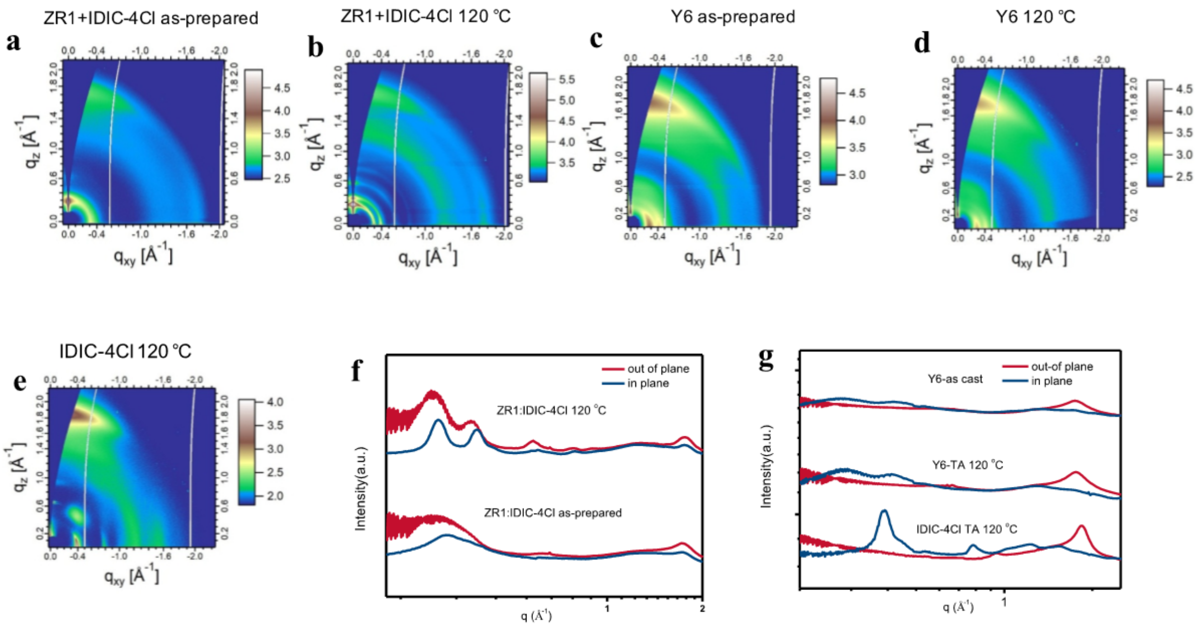

Supplementary Figure 6. (a–e) 2D GIWAXS patterns in pristine films and in blend films; (f-g) corresponding out-of-plane curves and in-plane curves.

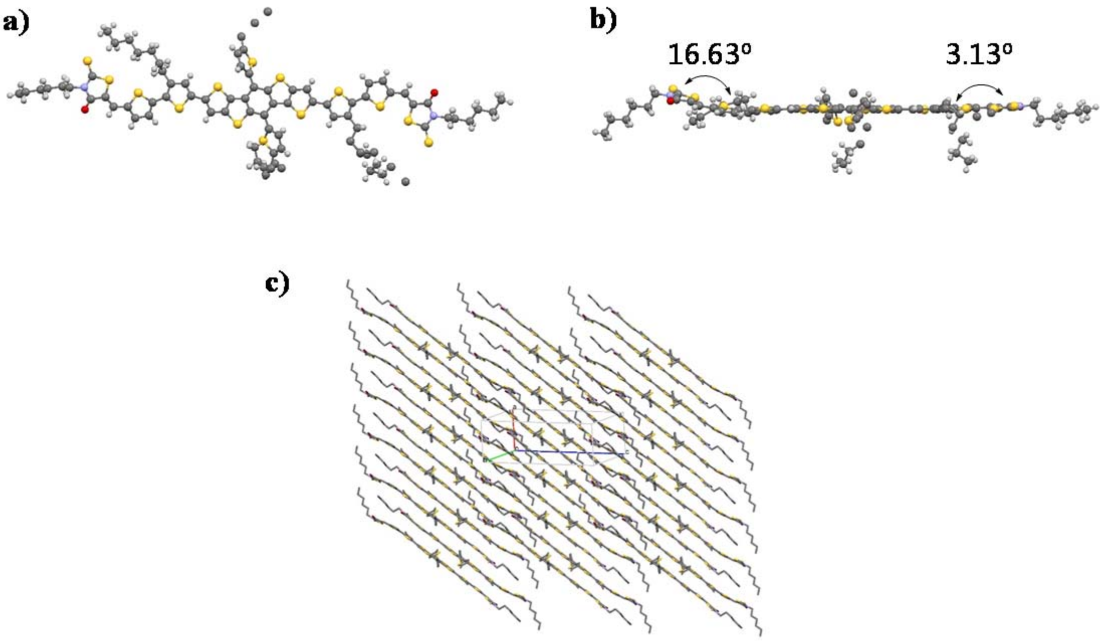

Supplementary Figure 7. Molecular conformation of ZR1 in the single crystal, top view (a) and side view(b), (c) crystal packing of ZR1 backbones in the (010) plane.

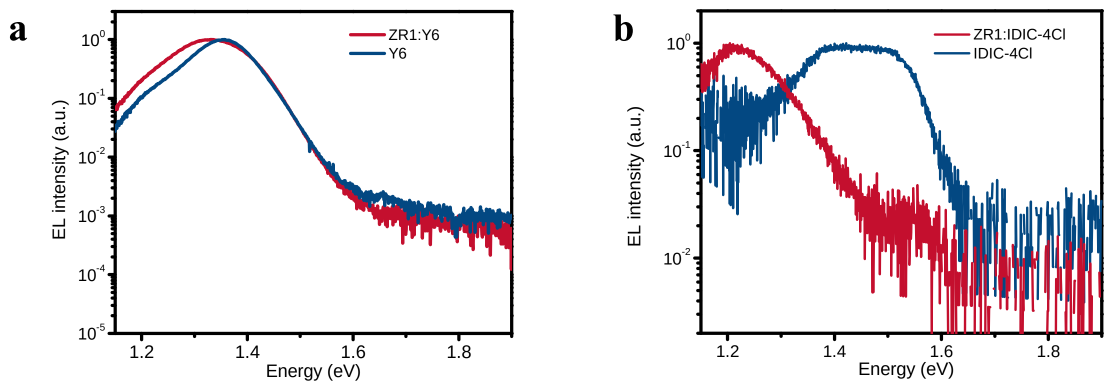

Supplementary Figure 8. (a–b) Electroluminescence spectrum of blend film and pure film.

Supplementary Tables

Supplementary Table 1. Optical properties and electrochemical of ZR1, Y6 and IDIC-4Cl.

**Compound**	$\lambda_{max}^{solution}$ (nm)	λ_{max}^{film} (nm)	$E_{g,film}^{opt}$ (eV)	**HOMO** ^{CV} (eV)	**LUMO** ^{CV} (eV)
**ZR1**	534	620	1.84	-5.32	-3.53
**IDIC-4Cl**	689	756	1.53	-5.91	-4.10
**Y6**	730	820	1.33	-5.72	-4.10

Supplementary Table 2. Device optimization of D:A ratios for ZR1: IDIC-4Cl at TA 120℃

**Donor/Acceptor**	**D:A**	V_{OC} **[V]**	J_{SC} **[mA cm^{-2}]**	**FF [%]**	**PCE [%]**
**ZR1:IDIC-4Cl**	1:0.6	0.789	17.14	66.37	9.08
**ZR1:IDIC-4Cl**	1:0.7	0.776	18.27	67.96	9.64
**ZR1:IDIC-4Cl**	1:0.8	0.785	17.53	66.03	9.20

Supplementary Table 3. Device optimization of thermal annealing temperature for ZR1: Y6 with D:A 1:0.5

**Donor/Acceptor**	**TA**	V_{OC} **[V]**	J_{SC} **[mA cm^{-2}]**	**FF [%]**	**PCE [%]**
**ZR1:Y6**	no	0.876	14.23	40.50	5.05
**ZR1:Y6**	110℃	0.870	24.02	66.20	13.62
**ZR1:Y6**	120℃	0.861	24.34	68.44	14.34
**ZR1:Y6**	140℃	0.837	24.49	67.90	13.91

Supplementary Table 4. Device optimization of D:A ratios for ZR1: Y6 at TA 120℃

**Donor/Acceptor**	**D:A**	V_{OC} **[V]**	J_{SC} **[mA cm**$^{-2}$**]**	**FF [%]**	**PCE [%]**
**ZR1:Y6**	1:0.4	0.855	20.56	67.07	11.80
**ZR1:Y6**	1:0.5	0.861	24.34	68.44	14.34
**ZR1:Y6**	1:0.6	0.869	21.18	62.24	11.46

Supplementary Table 5. Device optimization of solvent annealing with THF for ZR1: Y6 at TA 120℃

**Donor/ Acceptor**	**D:A**	V_{OC} **[V]**	J_{SC} **[mA cm**$^{-2}$**]**	**FF [%]**	**TA (°C)**	**SVA**	**PCE [%]**
**ZR1:Y6**	1:0.5	0.861	24.34	68.44	120	no	14.34
**ZR1:Y6**	1:0.6	0.851	22.46	62.58	120	THF (40s)	11.97

Supplementary Table 6. Device comparison of ZR1:Y6, ZR1-T:Y6, ZR1-3T:Y6 blends at TA 120℃

**Donor/Acceptor**	**D:A**	V_{OC} **[V]**	J_{SC} **[mA cm**$^{-2}$**]**	**FF [%]**	**PCE [%]**
**ZR1:Y6**	1:0.5	0.861	24.34	68.44	14.34
**ZR1-T:Y6**	1:0.6	0.498	0.0019	25.29	0.025
**ZR1-3T:Y6**	1:0.5	0.754	22.51	45.47	7.67

Supplementary Table 7. Detailed RSoXS of the ZR1: Y6 blend films under different condition

System	284.2eV circ.int	Thickness (nm)	SQRT (TSI/thickness)	Domain purity	Domain size (nm)
**ZR1:Y6 no TA**	6.65E-09	120	7.4434E-06	0.8752598	73.404
**ZR1:Y6 110 °C**	7.16E-09	120	7.72647E-06	0.9085459	72.359
**ZR1:Y6 120 °C**	7.99E-09	120	8.16236E-06	0.9598021	73.913
**ZR1:Y6 140 °C**	8.68E-09	120	8.50421E-06	1	83.412

Supplementary Table 8. Hole mobility and electron mobility of blend films and pure films.

	ZR1: Y6	ZR1: IDIC-4Cl	ZR1	Y6	IDIC-4Cl
**Hole mobility**	1.32×10^{-4}	3.30×10^{-4}	3.53×10^{-3}	-	-
**Electron mobility**	3.92×10^{-4}	4.55×10^{-4}	-	2.29×10^{-4}	1.08×10^{-4}

Supplementary Methods

Materials and synthesis Compounds 5 were purchased from Hyper Chemical Company, Compounds 4 and 8 were synthesized through the method described in the literature[1,2], And the detailed synthetic processes of the other monomers and target small molecules were illustrated as follows.

Compound 3: A solution of Compound 1 (4.30 g, 13.34 mmol), Compound 2 (2.12 g, 11.12 mmol) and Cs_2CO_3 (13.04g, 40.02mmol) in toluene (50 mL) was degassed by bubbling N_2 through the solution for 10 min. $Pd(PPh_3)_4$ (0.77 g, 0.68 mmol) was added and the resulting solution was vigourously refluxed overnight under inert atmosphere. The solution was cooled to room temperature and diluted with n-hexane, filtered and concentrated in vacuo. The crude product was purified by flash chromatography (SiO2, n-hexane, 5% EtOAc) to give Compound 3 (2.20 g, 7.19 mmol, 54% yield) as a yellow oil. ^{1}H NMR (400 MHz, $CDCl_3$) δ 9.88 (s, 1H), 7.69 (d, J = 3.7 Hz, 1H), 7.27 (s, 1H), 7.21 (d, J = 3.7 Hz, 1H), 6.97 (d, J = 5.1 Hz, 1H), 2.85-2.75(m, 2H), 1.70-1.60 (m, 2H), 1.41 – 1.20 (m, 10H), 0.87 (t, J = 6.4 Hz, 3H).

Compound 4: To a stirred solution of **3** (2.28 g, 7.44 mmol) in $CHCl_3$ (20 mL) and acetic acid (10 mL) kept in the dark at 0 ºC was added *N*-bromosuccinimide (1.32 g, 7.44 mmol). The solution was stirred before being stirred for 1.5 h, diluted with n-hexane and washed with water, $NaHCO_3$ (sat.) and brine. The organic layer was dried ($MgSO_4$) and concentrated in vacuo. The crude product was purified via flash chromatography (SiO_2, n-hexane, 0.5 – 6% EtOAc) to give Compound 4 (2.0 g, 5.2mmol, 70% yield) as a yellow oil. ^{1}H NMR (400 MHz, $CDCl_3$) δ 9.86 (s, 1H), 7.67 (d, J = 3.7 Hz, 1H), 7.12 (d, J = 3.6 Hz, 1H), 6.91 (s, 1H), 2.72 (t, J = 7.7 Hz, 2H), 1.63-1.56 (m, J = 15.1, 7.5 Hz, 2H), 1.39 – 1.19 (m, 10H), 0.87 (t, J = 6.3 Hz, 3H).

Compound 7: Compound 5 (500mg, 0.44 mmol) and compound 6 (511 mg, 1.32mol) were dissolved into anhydrous toluene (40 mL) in a three-neck flask. The solution was flushed with nitrogen for 20 min, and $Pd(PPh_3)_4$ (25.56 mg) was added into the solution quickly. Then, the solution was reacted at 110 °C for 72h under nitrogen protection. After cooling to room temperature, the toluene was removed under reduced pressure. The crude product was subsequently purified by column chromatography on silica gel (DCM) to afford compound c1 as red solid.^{1}H NMR (400 MHz, CDCl3) δ 9.90 (s, 2H), 7.71 (d, J = 3.9 Hz, 2H), 7.34 (s, 2H), 7.22 (t, J = 3.3 Hz, 4H), 7.05 (d, J = 3.3 Hz, 2H), 7.02 (s, 2H), 2.99 (d, J = 6.6 Hz, 4H), 2.83–2.77 (m, 4H), 1.82 (dd, J = 11.9, 6.0 Hz, 2H), 1.68 (dd, J = 15.3, 7.5 Hz, 4H), 1.52–1.24 (m, 52H), 0.90 (dt, J = 20.6, 6.7 Hz, 18H). MALDI-TOF MS (m/z): 1411.1

Compound ZR1: Compound 7 (200 mg, 0.142 mmol) and Compound 8 (307 mg, 1.4 mmol) was dissolved in dry $CHCl_3$ (30 ml). After 3 drops of piperidine were added, the resulting solution was refluxed and stirred for 12 h under nitrogen. After cooling to room temperature, the reaction mixture was poured into water and extracted several times with chloroform. The organic phases were combined, washed with brine, and dried over anhydrous magnesium sulfate. Then the solvent was removed under reduced pressure, and the crude product was purified by column chromatography on silica gel ($CHCl_3$) to yield Compound ZR1 as black solid. ^{1}H NMR (400 MHz, $CDCl_3$) δ 7.65 (s, 2H), 7.20 (s, 2H), 7.15 (d, *J* = 3.3 Hz, 2H), 7.06 (s, 2H), 6.99 (d, *J* = 6.2 Hz, 4H), 6.73 (s, 2H), 3.94 (t, *J* = 7.3 Hz, 4H), 3.02 (s, 4H), 2.72 – 2.61 (m, 4H), 1.92 – 1.80 (m, 2H), 1.62 (d, *J* = 5.3 Hz, 8H), 1.35 (t, *J* = 34.6 Hz, 64H), 1.03 – 0.81 (m, 24H).MALDITOF MS (m/z): 1810.6

Compound IDIC-4Cl: Compound 9 (300 mg, 0.0.39mmol) and compound 10 (1022mg, 7.77 mmol) were dissolved in dry $CHCl_3$ (60 ml). After 1 ml of pyridine was added, the resulting solution was

refluxed and stirred for 12 h under nitrogen. After cooling to room temperature, the reaction mixture was poured into 200 ml methanol and filtered. The crude product was purified by column chromatography on silica gel using petroleum ether/dichloromethane (1:1) as eluent to yield IDIC-4Cl as powder. ^{1}H NMR (400 MHz, $CDCl_3$) δ 9.00 (s, 2H), 8.79 (s, 2H), 7.96 (s, 2H), 7.75 (s, 2H), 7.63 (s, 2H), 2.08 (td, *J* = 13.0, 4.1 Hz, 4H), 1.96 (td, *J* = 13.0, 4.1 Hz, 4H), 1.20 (d, *J* = 7.2 Hz, 48H), 0.80 (t, *J* = 6.8 Hz, 12H). MALDI-TOF MS (m/z): 1260.9

Supplementary References

1 R. J. Kumar, J. M. MacDonald, T. B. Singh, L. J. Waddington, A. B. Holmes, *J. Am. Chem. Soc.* 2011, ***133***, 8564.

2 C. V. Kumar, L. Cabau, E. N. Koukaras, S. A. Siddiqui, G. D. Sharma, E. Palomares, *Nanoscale* 2015, ***7***, 7692.
